# Predictors of Suboptimal Adherence Among Children on Antiretroviral Therapy in Southern Ethiopia: A Multicenter Retrospective Follow-Up Study

**DOI:** 10.3389/ijph.2023.1606520

**Published:** 2023-11-09

**Authors:** Tamirat Gezahegn Guyo, Fasika Merid, Temesgen Mohammed Toma

**Affiliations:** Department of Public Health, Arba Minch College of Health Sciences, Arba Minch, Ethiopia

**Keywords:** antiretroviral therapy, children, HIV, suboptimal adherence, Ethiopia

## Abstract

**Objectives:** Despite increased access to and availability of antiretroviral therapy, the program’s effectiveness is primarily affected by treatment adherence. Therefore, this study aimed to determine the magnitude and predictors of suboptimal adherence among children on ART in Southern Ethiopia.

**Methods:** A multicenter retrospective study was conducted among human immunodeficiency virus (HIV) infected children in Gamo and South Omo zone public health facilities. To identify factors associated with suboptimal adherence, a binary logistic regression model was fitted. Variables with a *p*-value ≤0.25 in bivariable logistic regression analysis were included in multivariable logistic regression analysis. *p*-value <0.05 was used to declare statistical significance.

**Results:** The suboptimal adherence was determined to be 30.3% (95% CI: 25.5%, 35.6%). Advanced clinical stage, hemoglobin level <10 mg/dL, unchanged initial regimen, and non-disclosure of HIV sero-status were significant predictors of suboptimal adherence.

**Conclusion:** Suboptimal adherence is a significant public health problem in the study setting. Therefore, designing interventions towards improving adherence is needed especially for children with poor clinical characteristics.

## Introduction

Human immunodeficiency virus (HIV) remains a major global public health problem, having claimed 40.1 million lives so far with ongoing transmission in all countries globally. It is an infection that attacks the body’s immune system, and acquired immunodeficiency syndrome (AIDS) is the most advanced stage of the disease [1]. By the year 2020, about 1.7 million children under 15 years old were living with Human Immunodeficiency Virus (HIV) [2], and about 99,000 died of AIDS-related mortality worldwide [3]. Sub-Saharan Africa is the region most affected by HIV and about 90% of all adolescents and children living with HIV were living in the region [4]. Ethiopia is one of the Sub-Saharan African countries with a total of 43,400 children living with HIV, out of which 2,000 AIDS-related mortalities occurred by the year 2020 [5].

Adherence is described as “the extent to which a person’s behavior, such as taking medication, sticking to a diet, and/or implementing lifestyle changes, corresponds with agreed-upon recommendations from a healthcare provider” [6]. Any child who misses more than three dosages throughout a 1 month treatment term is considered to have less than 95% adherence, which is suboptimal adherence, and a child who misses a dose of fewer than three is considered to have optimal adherence [7]. The success and duration of ART drug regimens are mostly influenced by treatment adherence, which needs near-perfect adherence of up to 95% [8–10]. Suboptimal adherence among children remains a significant challenge for ART programs in both developed and developing countries [11]. According to evidence from studies conducted in numerous countries throughout the world, the extent of suboptimal adherence among HIV-positive children was expected to be substantial. In a systematic review conducted in low- and middle-income countries among children living with HIV, the level of adherence ranges from 49% to 100% [12]. Studies conducted in Sub-Saharan African countries showed that the adherence level was reported to be from 29% to 98% among children on ART [13–15]. Evidence from previous studies conducted in different parts of Ethiopia revealed that the magnitude of suboptimal adherence to ART ranged from 9.3% in Hossana [16] to 34% in Southwest Ethiopia [17].

Suboptimal treatment adherence is linked with an increase in the risk of unfavorable treatment outcomes like treatment failure and antiretroviral drug resistance, immunological decline resulting in opportunistic infections, and advanced HIV disease progression [18–22]. This in turn leads to preventable morbidity and mortality, increased expenditure for care, and avoidable forward transmission of HIV [23]. Poor adherence to ART reduces the effectiveness of viral suppression, increases viral resistance, and puts people living with HIV (PLHIV) at risk of hospital admission, and opportunistic infection [24]. Moreover, the effect of suboptimal adherence was also revealed by the outcome of the ambitious 90–90–90 targets, which aimed to attain 90% viral suppression. Due to this effect, only 40% of under-fifteen-year-old children living with HIV were virally suppressed [25].

Evidence from previous studies identified different factors associated with suboptimal treatment adherence of HIV-positive children on ART. These include the child’s age, the caregiver’s age, TB co-infection, advanced clinical stage, non-disclosure of children’s sero-status, and treatment failure [14, 26, 27].

Several strategies have been implemented to improve treatment adherence in children on ART, and they have shown benefits in improving adherence. Mobile phone text messages, behavioral skills training/medication adherence training for caregivers, fixed-dose combinations, and once-daily regimens are among the approaches [28]. Despite these efforts, poor adherence remains a significant hurdle to the efficacy of ART programs. The degree of suboptimal adherence level and factors associated with it can vary from place to place, so it is necessary to assess the problem in order to design effective strategies. However, there is limited evidence regarding suboptimal adherence to care among children on ART in Ethiopia, and no evidence in a study setting. Therefore, this study aimed to assess suboptimal adherence and associated factors among children receiving ART.

## Methods

### Study Design, Period, and Setting

An institution-based retrospective follow-up study was conducted from 12 April to 10 May 2022. The study was conducted in public health facilities found in Gamo, and South Omo zones, Southern Ethiopia. Arba Minch town is 505 Kilometers (KM) Southwest far from the capital city, Addis Ababa. Jinka town is about 563 KM far from Addis Ababa and 399 Kilometers from Hawassa. In these two zones, there are about 23 health facilities (2 general hospitals, 5 primary hospitals, and 16 health centers) that are currently providing pediatric ART services. These health facilities provide different services to the community in their catchment area and nearby woredas and zones. In these two zones currently, there are about 256 HIV-positive children on active ART follow-up. The follow-up schedule was based on the national ART guideline of Ethiopia; children beginning their follow-up returned for their first follow-up after 2 weeks of initiation of ART then monthly for the first 6 months, followed by every 3 months for drug refill, clinical assessment, and adherence support [23].

### Population

All HIV-positive children (<15 years) who were on ART in public health facilities of Gamo and South Omo Zones made up the source population. All HIV-positive children (<15 years) who were on ART in Jinka General Hospital, Arba Minch General Hospital, Gazer Primary Hospital, Chencha Primary Hospital, Birbir Health Center, Gerese Health Center, Jinka Millennium Health Center, Kamba Health Center, and Shele Health Center, from 1 January 2012 to 31 December 2021 made up the study population. All HIV-positive children (<15 years) who were on ART and had completed at least one follow-up visit were included in the study. Children on ART with incomplete records were excluded from the study.

### Sample Size Determination and Sampling Technique

#### Sample Size Determination

The sample size was determined by using a single population proportion formula [n = [(Z_a/2_)^2^*P (1−P)]/d^2^] and by considering a 95% confidence interval with a confidence level of Z_a/2_ = 1.96, the proportion of sub-optimal adherence of 21.8% taken from a study conducted in south Gondar public hospitals, Northwest Ethiopia [26], a margin of error (d) 5%. Using the above assumptions, the calculated sample size was 262 and after adding a 10% incompleteness rate, the final sample size was 289.

#### Sampling Technique and Procedure

The public health facilities in Gamo and South Omo Zones were stratified based on their type of health facility into General Hospitals, Primary Hospitals, and Health Centers. Then by simple random sampling (lottery method) two Primary Hospitals and five Health Centers were selected. Both of the General Hospitals were included. The children <15 years of age were identified in each of the health facilities using medical record numbers (MRN) that were obtained from electronic databases. Patients’ charts were drawn using the MRN. In these health facilities 349 children (age < 15) on ART from 01 January 2012 to 31 December 2021 were identified and 323 of the children who fulfilled the eligibility criteria within the follow-up period were included in the study.

### Data Collection Tools and Procedures

Data were collected by using a data extraction checklist developed in English from the standardized ART intake and follow-up forms from national HIV guideline [23], and by reviewing related literatures. The tool contains socio-demographic, clinical, and treatment-related characteristics of participants. The lists of study participants were taken from the ART data clerk by using children’s MRN or unique ART numbers. Charts of the children were taken from card rooms. Then data were collected by reviewing the patient follow-up charts by fourteen data collectors and three supervisors.

### Operational Definitions

Suboptimal adherence: If HIV-positive children on ART experienced fair or poor adherence (drug adherence of ≤94% or ≥3 doses missed monthly) [23].

CD4 count for severe immunodeficiency: The classification was based on children’s age. For children < 5 years CD4 < 200 cells/mm^3^, and CD4 < 100 cells/mm^3^ children ≥ 5 years [29].

Nutritional status: Was measured by weight for age (WFA) and body mass index (BMI) for Age. Categorized as; underweight (WFA or BMI for age <−z-score) and normal (z-score > −2) [23].

### Data Quality Assurance

The data collection process was conducted by 14 data collectors (9 BSc in Nursing and 5 BSc in Public Health), who were trained on comprehensive HIV care and providing follow-up care services, and three supervisors (BSc in Public Health) after receiving 1 day of training. To check the relevance, consistency, and adequacy of the checklist, a pretest was conducted before the actual data collection in the same setting. The data collection tool was properly numbered and coded. The principal investigator and supervisors carried out daily monitoring of the data collection process by reviewing and checking the filled-out checklists to ensure accuracy, completeness, and consistency.

### Data Processing and Statistical Analysis

The collected data were entered into Epi-Data version 3.1 and then exported to STATA version 14.0 for management and analysis. Exploratory data analysis was done to check the presence of potential outliers, normality, and level of missing values. Z-scores, to assess nutritional status, were generated by using WHO Anthro-Plus software. Descriptive statistics were found using mean, median, standard deviation, interquartile range, frequencies, and percentages. A bivariable logistic regression model was fitted to assess the association between each independent variable and the dependent variable, variables with a *p*-value ≤ 0.25 in bivariable logistic regression were candidates for multivariable analysis. A multivariable logistic regression model with a backward likelihood ratio method was fitted to identify factors significantly associated with suboptimal adherence. Multicollinearity was checked by using variance inflation factor (VIF) and tolerance, the mean VIF = 1.08, indicating no threat of collinearity. The goodness of fit of the model was checked by the Hosmer-Lemeshow chi-square test (Prob > X^2^ = 0.2309). AOR with a 95% CI and corresponding *p*-value was used to identify statistically significant variables. *p*-value <0.05 was used to declare statistical significance.

## Result

From a total of 349 children (age <15 years) who were receiving ART from 1st January 2012 to 31st December 2021, about 323 have fulfilled the inclusion criteria and are included in the analysis with a 92.6% completeness rate of charts.

### Sociodemographic Characteristics of Children Receiving ART

The median age of the study participants was 5 (IQR: 2–9) years, 118 (36.5%) children were in the age group of ≤3 years, and approximately a quarter (91 (28.2%)) of the participants were in the age group of ≥9 years. Among the study participants, 167 (51.7%) were male and forty-five children (13.9%) had lost both of their parents. For the majority of the children, 277 (85.8%) caregivers were their biological families. Three percent of the caregivers were in the age range of ≤24 years and 135 (41.8%) of the caregivers were in the age group of 35–44 years. Regarding the place of residence, more than half (50.8%) of the HIV-positive children were urban residents. Half of the caregivers [165 (51.1%)] had not attended formal education and 28 (8.6%) attained a tertiary and above educational level. One-third of the caregivers [112 (34.6%)] were daily laborers and 42 (13%) were government employees (Table 1).

**TABLE 1:** Socio-demographic characteristics of children on antiretroviral therapy in public health facilities of Gamo and South Omo zones, southern Ethiopia, 2022 (*n* = 323) (Predictors of Suboptimal Adherence Among Children on Antiretroviral Therapy in Southern Ethiopia, 2022: A Multicenter Retrospective Follow-Up Study).

Characteristics	Categories	Frequency (*n*)	Percent (%)
Age (in years)	≤3	118	36.5
	4–8	114	35.2
	≥9	91	28.2
Sex	Male	156	48.3
	Female	167	51.7
Residence	Urban	164	50.8
	Rural	159	49.2
Parent status	Both alive	144	44.6
	Either died	134	41.4
	Both died	45	13.9
Caregiver-child relationship	Biological family	277	85.8
	Others^a^	46	14.2
Age of caregiver	≤24 years	12	3.7
	25–34 years	112	34.6
	35–44 years	135	41.8
	≥45 years	64	19.8
Educational status of caregiver	No formal education	165	51.1
	Primary	105	32.5
	Secondary	25	7.7
	Tertiary and above	28	8.6
Occupational status of caregiver	Farmer	87	26.9
	Merchant	51	15.8
	Gov’t employee	42	13
	Daily laborer	112	34.6
	Others^b^	31	9.6

Abbreviation: ART, antiretroviral therapy.

Note: ^a^Other: relative, not relative.

^b^ Others: Housewife, Gospel preacher.

### Clinical and Treatment-Related Characteristics of Children on ART

Among the 323 HIV-positive children who participated in the study, more than three-quarters (78.0%) were from hospitals, and the remaining 71 (22.0%) were from health centers. One hundred sixty-four (50.8%) HIV-positive children were enrolled with a test-and-treat approach. Regarding the BMI for age z-score, 222 (68.7%) of the study participants had a BMI for age z-score of ≥−2. Regarding baseline weight for age and height for age z-scores, 33.6% and 32.5% of children had a z-score of <−2, respectively. Among the under-five children who participated in the study, 99 (65.1%) had reached a developmental milestone that was appropriate for their age, whereas 53 (34.8%) had a delayed or regressed developmental milestone. The functional status of the HIV-positive children aged above 5 years was identified to be 49.1%, 45.6%, and 5.3%, labeled as working, ambulatory, and bedridden, respectively. More than eighty percent of the participants had a hemoglobin level of ≥10 mg/dL and the mean hemoglobin level was 12.5 mg/dL (2.45 mg/dL SD). Concerning the WHO clinical stage, of the total children who participated in the study, 209 (64.7%) were in clinical stage I or II. The study reported that 45 (13.9%) HIV-positive children had a history of TB/HIV co-infection. In addition, 68 (21.1%) of the participants had a history of opportunistic infections other than TB. For the prevention of common opportunistic infections, 157 (48.6%) of the children on ART took both CPT and IPT. The median baseline CD4 count was determined to be 473 (IQR: 288–783) cells/mm^3^. Regarding ART regimens initiated during enrollment, 172 (53.3%) were started on Zidovudine, Lamivudine, and Nevirapine (AZT+3TC+NVP)-containing regimens, and only 21 (6.5%) were initiated on Dolutegravir (DTG)-containing regimens. The HIV sero-status of 72 (22.3%) children on ART was disclosed to the children themselves. The initial ART regimen of 149 (46.1%) children was not changed, and the children were taking their old ART regimen. Availability of new drugs was the main reason for regimen change, but other reasons, such as treatment failure, drug stockouts, and other unknown reasons, were also responsible. Only four children on ART experienced side effects.

### Predictors of Suboptimal Treatment Adherence

**FIGURE 1:**
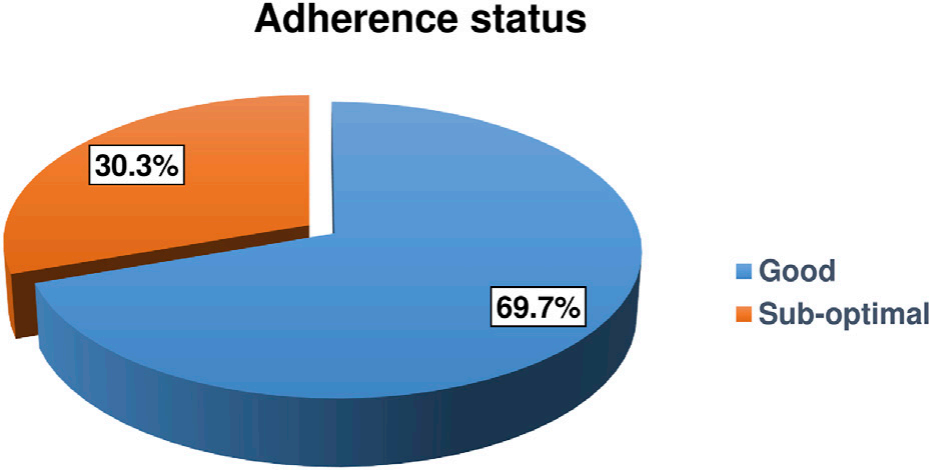
Adherence status of HIV-positive children on antiretroviral therapy in Gamo and South Omo zones public health facilities, Southern Ethiopia, 2022 (*n* = 323) (Predictors of Suboptimal Adherence Among Children on Antiretroviral Therapy in Southern Ethiopia, 2022: A Multicenter Retrospective Follow-Up Study).

This study identified that the magnitude of suboptimal adherence was 30.3% (95% CI: 25.5%, 35.6%) (Figure 1).

In bivariable logistic regression analysis, suboptimal adherence was significantly associated with; sex of the child, parent status, educational status of caregiver, type of health facility, BMI for age, WHO clinical stage, history of tuberculosis infection, CPT prophylaxis, IPT prophylaxis, hemoglobin level, CD4 count, regiment change, and disclosure status at *p*-value of <0.25. In multivariable logistic regression analysis, WHO clinical stage, hemoglobin level, regimen change, and disclosure status of the children showed statistically significant association with suboptimal adherence at *p*-value <0.05.

The odds of suboptimal adherence was doubled (AOR = 2.29; 95% CI: 1.13, 3.98) among children with a WHO clinical stage of III or IV when compared to those with a WHO clinical stage of I or II. Children receiving ART with hemoglobin levels below 10 mg/dL had 3.62 times increased odds of suboptimal adherence when compared to their counterparts (AOR = 3.62; 95% CI: 1.82, 7.19). The odds of suboptimal adherence was nearly three times higher among children whose initial regimen was not changed when compared to those children whose initial regimen was changed (AOR = 2.69; 95% CI: 1.55, 4.67). Children whose HIV sero-status was disclosed had 2.46 times increased odds of suboptimal adherence when compared to their counterparts (AOR = 2.46; 95% CI: 1.17, 5.19) (Table 2).

**TABLE 2:** Bivariable and multivariable analysis for predictors of suboptimal adherence among children on antiretroviral therapy in public health facilities of Gamo and South Omo zones, Southern Ethiopia, 2022 (*n* = 323) (Predictors of Suboptimal Adherence Among Children on Antiretroviral Therapy in Southern Ethiopia, 2022: A Multicenter Retrospective Follow-Up Study).

Variables	Categories	Suboptimal adherence		COR (95% CI)	AOR (95% CI)	*p*-value
		Yes (%)	No (%)			
Sex	Male	41 (12.7)	115 (35.6)	1	1	
	Female	57 (17.7)	110 (34.1)	1.45 (0.90, 2.35)	1.68 (0.99, 2.88)	0.06
Parent status	Both alive	39 (12.1)	105 (32.5)	1	1	
	Either died	48 (14.9)	86 (26.6)	1.50 (0.90, 2.50)	1.35 (0.75, 2.42)	0.32
	Both died	11 (3.4)	34 (10.5)	0.87 (0.40, 1.89)	1.03 (0.43, 2.48)	0.95
Educational status of the caregiver	No formal education	64 (19.8)	101 (31.3)	2.72 (1.28, 5.80)	2.06 (0.90, 4.74)	0.09
	Primary	24 (7.4)	81 (25.1)	1.27 (0.56, 2.91)	1.16 (0.47, 2.83)	0.75
	Secondary and above	10 (3.1)	43 (13.3)	1	1	
Type of health facility	Hospital	82 (25.4)	170 (52.6)	1	1	
	Health center	16 (5.0)	55 (17.0)	0.60 (0.33, 1.12)	1.04 (0.46, 2.35)	0.92
BMI for age	z-score < −2	36 (11.2)	65 (20.1)	1.43 (0.87, 2.36)	1.08 (0.60, 1.93)	0.80
	z-score ≥ −2	62 (19.2)	160 (49.5)	1	1	
WHO clinical stage	I or II	47 (14.6)	162 (50.2)	1	1	
	III or IV	51 (15.8)	63 (19.5)	2.79 (1.71, 4.56)	2.29 (1.31, 3.98)	0.003
Presence of TB	Yes	24 (7.4)	21 (6.5)	3.15 (1.66, 5.99)	1.75 (0.80, 3.83)	0.16
	No	74 (22.9)	204 (63.2)	1	1	
CPT Prophylaxis	Yes	86 (26.6)	167 (51.7)	1	1	
	No	12 (3.7)	58 (18.0)	0.40 (0.20, 0.79)	0.98 (0.43, 2.22)	0.96
IPT Prophylaxis	Yes	56 (17.3)	158 (48.9)	1	1	
	No	42 (13.0)	67 (20.7)	1.77 (1.08, 2.89)	1.21 (0.62, 2.37)	0.58
Hemoglobin level	<10 mg/dL	34 (10.5)	18 (5.6)	6.11 (3.23, 11.54)	3.62 (1.82, 7.19)	<0.001
	≥10 mg/dL	64 (19.8)	207 (64.1)	1	1	
CD4 count	≤200 cells/mm^3^	30 (9.3)	31 (9.6)	3.35 (1.84, 6.07)	1.49 (0.70, 3.17)	0.30
	200–350 cells/mm^3^	20 (6.2)	28 (8.7)	2.47 (1.28, 4.77)	1.76 (0.81, 3.82)	0.15
	≥350 cells/mm^3^	48 (14.9)	166 (51.4)	1	1	
Regimen change	Yes	37 (11.5)	137 (42.4)	1	1	
	No	61 (18.9)	88 (27.2)	2.57 (1.57, 4.18)	2.69 (1.55, 4.67)	<0.001
Disclosure status	Disclosed	11 (3.4)	61 (18.9)	1	1	
	Not disclosed	87 (26.9)	164 (50.8)	2.94 (1.47, 5.88)	2.46 (1.17, 5.19**)**	0.018

Abbreviations: ART, antiretroviral therapy; BMI, body mass index; CD4, cluster of differentiation 4; CPT, cotrimoxazole preventive therapy; IPT, isoniazid preventive therapy; WHO, world health organization.

## Discussion

This retrospective follow-up study was conducted to identify the predictors of suboptimal adherence among children on ART. As a result, advanced clinical stage (III or IV), low hemoglobin level, unchanged regime, and non-disclosure of HIV sero-status of the children were identified to be the independent predictors of suboptimal adherence.

This study revealed that the magnitude of suboptimal adherence was found to be 30.3%. This finding is lower than studies conducted in Dar es Salaam [14] and Northern Tanzania [30] which reported a magnitude of 40% and 75.4%, respectively. The result of this study is in line with the studies conducted in New Delhi [31] (34.4%) Uganda [32] (28.05%) Gondar in Ethiopia [33] (31.9%) and Ambo in Ethiopia [34] (33.3%). On the contrary, the magnitude of suboptimal adherence determined by this study was reported to be higher than previous studies conducted in South India [35] (9.1%) and Nigeria [36] (14%). Likewise, it was higher than studies previously conducted in different parts of Ethiopia which reported a magnitude of suboptimal adherence ranging from 5.16% to 21.8% among HIV-positive children who are on ART [26, 37–40].

The disparity could be attributed to differences in the adherence level diagnosis technique, the socio-demographic and cultural backgrounds of study participants, and patient-healthcare provider relationships in various settings. Socioeconomic status, study design, adherence assessment approaches, sample size, and setting differences may also be factors. Furthermore, under or over-reporting is more likely in low-income nations than in middle-income countries due to a shortage of expertise among healthcare personnel or caregivers. The consistency may be due to similarity in data recording formats and follow-up charts in the ART program for pediatric HIV Care and treatment in Ethiopia which was prepared by the Federal Minister of Health of Ethiopia [41].

In this study, the WHO clinical stage was associated with suboptimal treatment adherence among HIV-positive children on ART. Children with advanced clinical stage (stage III or IV) had twice the increased risk of suboptimal adherence when compared to those with a WHO clinical stage I or II. This is consistent with a study conducted in Dar es Salaam, Tanzania [14]. However, this result of the current studies is inconsistent with studies conducted in Ethiopia [34, 37, 42] which stated that children with advanced clinical stages (III/IV) were more likely to adhere. This may be due to the fact that children at advanced WHO clinical stages of the disease may be hopeless for their survival and hence may give little attention or even be ignorant of their medication as compared to their counterparts that are at stage I or II.

The current study reported that children receiving ART with hemoglobin levels below 10 mg/dL had 3.62 times increased odds of suboptimal adherence when compared to their counterparts. This is likely because of the exacerbation of previously existing anemia among children who started on a Zidovudine (AZT)-containing regimen, which can further lead to additional opportunistic infections and in turn reduce adherence due to the tension of the concurrent illness. In addition, it can also be because of the reduction in ART tolerance due to decreased absorption and the effect of immune defense from anemia.

Moreover, regimen change was another factor associated with suboptimal adherence, as identified by this study. The odds of suboptimal adherence was nearly three times higher among children whose initial regimen was not changed when compared to those children whose initial regimen was changed. This is consistent with a study done in Northwest Ethiopia [26] that states that children enrolled in the Zidovudine-containing ART regimen were more likely to be poor adherents. This regimen is the oldest one and is currently replaced with a Dolutegravir-containing regimen; hence, children on this old regimen have not had their regimen changed. This may be due to the fact that the majority of the old ART regimens have side effects that enhance the progression of the disease and result in subsequent complications. The Zidovudine-containing ART regimen was associated with anemia that imposed additional effects on the immune system.

Furthermore, the disclosure status of HIV-positive children on ART was found to be associated with suboptimal adherence, which is controversial. Therefore, children whose HIV sero-status was disclosed had 2.46 times higher odds of suboptimal adherence when compared to their counterparts. This is consistent with studies conducted in Uganda [32], Ethiopia [27, 33], and on the other hand, the report from the current study contradicts a study done in Tikur Anbessa Hospital, Ethiopia [42] that states that HIV-positive children who were not aware of their HIV sero-status were more likely to adhere. Children who are not aware of their HIV status may not grasp the rationale for taking drugs and may become resistant to them since they do not understand why they take medicine while appearing to be well. Children who are aware of their HIV sero-status may feel doomed, refuse, and intentionally miss the treatment doses until they accept their HIV sero-status, and the observed variation between studies may be associated with the extent and duration of counseling provided to children.

The study used a long year of data to identify predictors of suboptimal adherence among the children receiving ART. It is a retrospective study and used secondary data, the effect of some variables (like socioeconomic status and viral load) was not assessed because of incomplete recoding. Since the data were collected at a point in time retrospectively; it was difficult to ascertain potential cause-effect or temporal relationship.

### Conclusion

The magnitude of suboptimal adherence was found to be high in the study settings. Advanced clinical stage, low hemoglobin level, unchanged regimen, and non-disclosure of HIV sero-status were the identified predictors of suboptimal adherence. To improve treatment adherence, special attention should be given to adherence counseling and training of adherence supports, especially for children with poor baseline clinical characteristics, those who are taking old regimens, and children with non-disclosed HIV sero-status. Researchers should conduct a longitudinal prospective study to address further clinical and Sociodemographic predictors of suboptimal adherence.
